# Olfactory instruction for fear: neural system analysis

**DOI:** 10.3389/fnins.2015.00276

**Published:** 2015-08-06

**Authors:** Newton S. Canteras, Eloisa Pavesi, Antonio P. Carobrez

**Affiliations:** ^1^Department of Anatomy, Institute of Biomedical Sciences, University of São PauloSão Paulo, Brazil; ^2^Department of Pharmacology, CCB, Federal University of Santa CatarinaFlorianópolis, Brazil

**Keywords:** predator odor, innate fear, fear conditioning, hypothalamic circuits, amygdala

## Abstract

Different types of predator odors engage elements of the hypothalamic predator-responsive circuit, which has been largely investigated in studies using cat odor exposure. Studies using cat odor have led to detailed mapping of the neural sites involved in innate and contextual fear responses. Here, we reviewed three lines of work examining the dynamics of the neural systems that organize innate and learned fear responses to cat odor. In the first section, we explored the neural systems involved in innate fear responses and in the acquisition and expression of fear conditioning to cat odor, with a particular emphasis on the role of the dorsal premammillary nucleus (PMd) and the dorsolateral periaqueductal gray (PAGdl), which are key sites that influence innate fear and contextual conditioning. In the second section, we reviewed how chemical stimulation of the PMd and PAGdl may serve as a useful unconditioned stimulus in an olfactory fear conditioning paradigm; these experiments provide an interesting perspective for the understanding of learned fear to predator odor. Finally, in the third section, we explored the fact that neutral odors that acquire an aversive valence in a shock-paired conditioning paradigm may mimic predator odor and mobilize elements of the hypothalamic predator-responsive circuit.

## General concepts regarding predator odor and its impact on defensive behavior research

Olfactory cues are effective stimuli for the retrieval of stressful memories (Wiemers et al., 2014). Notably, when exposed to a trauma-associated olfactory cue, post-traumatic stress disorder-vulnerable victims have been shown to exhibit clinical symptoms in addition to increased regional blood flow in several regions related to fear and anxiety processing, including the amygdala, insula, medial prefrontal cortex, and anterior cingulate cortex (Vermetten et al., 2007). Thus, understanding of the neural circuits involved in fear encoding and expression in response to an olfactory stimulus seems to be an important step for translational studies that are attempting to uncover the neural mediation of pathological fear memory encoding in anxiety disorders.

Predator odors are semiochemical cues that evolved and were incorporated as species memory, or phyletic memory (Fuster, 1997). For this reason, regardless of any previous contact with a real cat, lab rodents will promptly recognize a cat odor and exhibit defensive behavior, such as increased immobility and the stereotyped stretched attend/approach postures that are characteristic of risk assessment behavior (Van Der Poel, 1979; Pinel et al., 1989). These instinctive fear responses are also present when subjects are re-exposed to a predator odor-associated environment, suggesting the acquisition and consolidation of contextual memory to predator odor (Hubbard et al., 2004).

Cat odor has been used as a valuable tool to study fear and anxiety. Compared to live cat exposure, cat odor exposure induces less intense instinctive fear responses, which can be reduced by anxiolytic drugs and are generally related to the approach-avoidance conflict—a general hallmark in anxiety (Blanchard et al., 1990, 1993; Zangrossi and File, 1992; Dielenberg and Mcgregor, 2001). However, it is difficult to standardize the amount of cat odor stimulus presented, and different degrees of freezing and avoidance may be found depending on the strength of the stimulus (Takahashi et al., 2005). When a hiding place is available, cat odor exposure increases the time the animal spends in an enclosed compartment and the time spent in the stretched-out posture within the enclosed compartment, which serves as an index of risk assessment behavior (Mcgregor et al., 2002; Dielenberg et al., 2004). Using a similar paradigm, Do Monte et al. (2008) found comparable defensive responses during exposure to the cat odor-associated context on the day following cat odor exposure, including an increased duration of time spent in the enclosed compartment and in stretched-out behavior.

A great deal has been learned about the olfactory pathways that mediate defensive responses to a number of predator odors (Takahashi, 2014; Pérez-Gómez et al., 2015). Of particular relevance for the present review, a number of the other predator odors that have been tested, excluding TMT (2,5-dihydro-2,4,5-trimethylthiazole), which is present in fox feces, have been shown to engage neural elements that are also responsive to cat odor (Pérez-Gómez et al., 2015). This finding suggests that multiple parallel mechanisms for the detection of different predator odors seem to converge in the brain to facilitate a common behavioral response. In this way, cat odor may be used as a good model to understand the neural mediation of defensive responses to other types of predator odor. In the present review, we will focus on three lines of work examining the dynamics of the neural systems that organize innate and learned fear responses to cat odor. In the first section, we will explore the neural systems involved in innate and learned fear responses to cat odor by examining the role of glutamatergic and beta-adrenergic transmission in the expression of innate fear and in the acquisition of contextual fear learning, with particular emphasis on the role of the dorsal premammillary nucleus (PMd) and the dorsolateral periaqueductal gray (PAGdl) as key sites that influence innate fear and contextual conditioning. In the second section, we will explore how chemical stimulation of these critical sites (i.e., the PMd and PAGdl) may be used as a useful unconditioned stimulus (US) in an olfactory fear conditioning paradigm and is likely to mimic predatory threats in instructing prosencephalic sites of higher order processing. Finally, in the third section, we will explore our findings regarding the use of a neutral odor as a conditioned stimulus (CS) in shock-based fear conditioning and determine how elements of the hypothalamic circuit that are responsive to predator odor may be engaged in the expression of shock-based olfactory fear conditioning.

## Neural mediation of innate fear responses to predator odor

**Figure 1:**
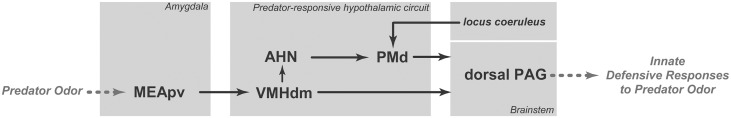
**Schematic diagram showing the putative brain circuit involved in organizing innate defensive responses to predator odor**. AHN, anterior hypothalamic nucleus; MEApv, medial amygdalar nucleus, posteroventral part; PAG, periaqueductal gray; PMd, dorsal premammillary nucleus; VMHdm, ventromedial hypothalamic nucleus, dorsomedial part. See text for discussion.

Predator odors are thought to work as kairomones, which are semiochemicals that are released by one species and have a favorable adaptive effect on a different “receiving” species but no favorable effect on the transmitting species (Dicke and Grostal, 2001; Wyatt, 2014). A number of studies have shown that the detection of predator odors relies on distinct olfactory subsystems for chemodetection, namely the vomeronasal organ (VNO) (McGregor et al., 2004), the Grueneberg ganglion (GG) (Brechbuhl et al., 2013), and subsets of sensory neurons within the main olfactory epithelium (MOE) that express trace amine-associated receptors (TAARs) (Liberles, 2015). Nasal detection of different predator odors has been associated with distinct olfactory subsystems, i.e., 2-phenylethylamine, which is found in carnivore urine, activates TAAR4 neurons in the MOE (Ferrero et al., 2011; Dewan et al., 2013), cat fur odor activates the VNO (McGregor et al., 2004), and 2-propylthietane, which is extracted from the stoat anal gland, is a GG activator (see Pérez-Gómez et al., 2015). Exposure to all of these different types of predator odors results in increased Fos expression in the posteroventral part of the medial amygdalar nucleus (MEApv) and in the dorsomedial part of the ventromedial hypothalamic nucleus (VMHdm; see Pérez-Gómez et al., 2015). Thus, an important concept emerges from this analysis: the detection of different predator odors converges in a pathway formed by the MEApv and VMHdm (Figure 1). The MEApv is part of the vomeronasal amygdala and is known to provide dense input to the VMHdm (Canteras et al., 1995), which, in turn, plays a critical role in the integration of predator-related defensive responses.

Optogenetic activation of steroidogenic factor 1 (SF1)-expressing neurons in the dorsomedial and central parts of the VMH (VMHdm/c) initiates a range of context-dependent somatomotor and autonomic responses that resemble animals' natural defensive behaviors (Wang et al., 2015). As shown by Wang et al. (2015), during VMHdm/c stimulation, if a hiding place was available, animals went to the hiding box and stayed inside of the box despite continued stimulation. Conversely, in the absence of the hiding box, VMHdm/c stimulation evoked either freezing or running and jumping, depending on the intensity of the stimulation. Moreover, VMHdm/c stimulation promoted avoidance, and the animals tended to avoid the place where they had received the stimulation. Taken together, these results indicate that VMHdm/c stimulation can induce complex defensive behavioral responses, including immobility, escape jumping, hiding, and avoidance. The main targets of the VMHdm/c are the dorsal periaqueductal gray (dPAG) and the anterior hypothalamic nucleus (AHN; Figure 1). Activation of the VMHdm/c projection to the dPAG induces inflexible immobility, whereas stimulation of the pathway from the VMHdm/c to the AHN promotes avoidance as well as immobility, running, and escape jumping (Wang et al., 2015). Thus, the direct pathway to the dPAG may be responsible for a rapid freezing response upon detection of a predator, whereas the AHN pathway may integrate a more complete range of complex defensive responses.

At least a portion of the complex defensive behaviors that have been described for the AHN are likely to be mediated through its projections to the dorsal premammillary nucleus (PMd; Figure 1). The AHN provides a dense bilateral projection to the PMd (Risold et al., 1994), which is the most responsive brain site to a predator or its odor (Canteras et al., 1997; Dielenberg et al., 2001; Cezario et al., 2008). Notably, PMd lesions have been shown to be highly effective in reducing anti-predator defensive responses (Canteras et al., 1997; Blanchard et al., 2003; Cezario et al., 2008). Pharmacological blockade of the PMd has also been shown to reduce innate defensive responses to cat odor (Canteras et al., 2008; Do Monte et al., 2008). In fact, the AHN, VMHdm and PMd form a partially segregated circuit in the medial zone of the hypothalamus; this circuit has been coined the predator-responsive circuit (Gross and Canteras, 2012). The PMd is likely to amplify neural processing in the predator-responsive hypothalamic circuit (Cezario et al., 2008). In line with this view, the PMd is particularly responsive to predator cues, and lesions or pharmacological blockade of this region drastically reduce anti-predator defensive responses. Conversely, PMd lesions are ineffective in other threatening situations, such as the elevated plus maze and exposure to post-shock contextual cues, suggesting the PMd may play a differential role in anti-predatory defensive responses (Blanchard et al., 2003).

The PAG is the major target of the predator-responsive circuit; the PMd and the VMHdm, albeit to a lesser extent, provide extensive projections to the dorsolateral and dorsomedial parts of the PAG. The projections are mostly aimed at the rostral and intermediate rostro-caudal levels of the PAG, and at caudal levels of the PAG, a moderate input is also centered in the lateral and ventrolateral divisions (Canteras, 2002). Notably, the spatial distribution of the axonal projections from the predator-responsive circuit to the PAG closely matches the pattern of Fos activation elicited in the PAG following exposure to predator odor (Dielenberg et al., 2001). Thus, rats exposed to cat odor present a particularly large increase in Fos activation in the dorsomedial and dorsolateral regions of the rostral two-thirds of the PAG in addition to somewhat sparser Fos labeling in the lateral and ventrolateral PAG at caudal levels (Dielenberg et al., 2001). The PAGdl seems to be critical for the integration of forebrain limbic information related to predator odor. On the efferent side, the PAGdl appears to control the entire range of defensive responses to predator odor, including flight, immobility, hiding and risk assessment behaviors (Blanchard et al., 1990; Carrive, 1993; Keay and Bandler, 1993). In support of this view, lesions in the dorsal PAG have also been shown to block all types of fear responses to predator odor (Sukikara et al., 2010).

Moreover, exposure to cat odor up-regulates Fos expression in the locus coeruleus (Dielenberg et al., 2001; McGregor et al., 2004). Firing rates in the locus coeruleus increase during stressful situations, and the locus coeruleus has been associated with fear responses (Bremner et al., 1996; Schenberg et al., 2001). Importantly, locus coeruleus lesions decrease both unconditioned and conditioned fear responses (Neophytou et al., 2001). In line with this view, systemic administration of propranolol (a centrally acting beta-blocker) reduced unconditioned defensive behaviors and PMd Fos expression in response to cat odor (Do Monte et al., 2008). This effect of noradrenergic transmission on fear responses should be, at least in part, mediated by noradrenergic projections from the locus coeruleus to the PMd (Sobrinho and Canteras, 2011; Figure 1), where beta-adrenoceptor blockade before cat odor exposure has been shown to reduce defensive responses to cat odor (Do Monte et al., 2008).

In summary, as shown in Figure 1, predator odors are processed by the MEApv, which engages the medial hypothalamic predator-responsive circuit. The predator-responsive circuit is further modulated by significant noradrenergic input from the locus coeruleus (which likely plays a critical role in the response to predator odors) and projects to the dorsal PAG, which is involved in the expression of innate defensive responses.

## Neural mediation of learned fear responses to predator odor

Exposure to a predator or its odor evokes robust contextually conditioned defensive responses (Blanchard et al., 2001; Mcgregor et al., 2002). Contextual defensive responses are characterized by freezing, risk assessment behavior, and avoidance of the environment where a predator or its odor had been previously encountered.

**Figure 2:**
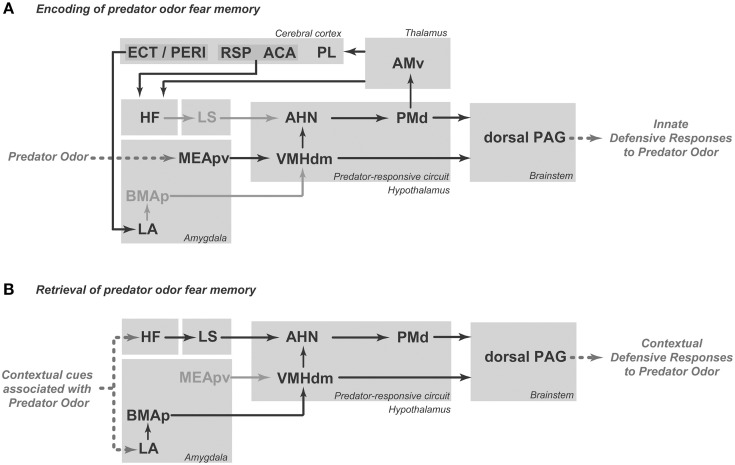
**Schematic diagrams showing the putative brain systems that support the encoding (A) and retrieval (B) of learned fear to predator odor**. ACA, anterior cingulate area; AHN, anterior hypothalamic nucleus; AMv, anteromedial thalamic nucleus, ventral part; BMAp, basomedial amygdalar nucleus, posterior part; ECT, ectorhinal area; HF, hippocampal formation; LA, lateral amygdalar nucleus; LS, lateral septum; MEApv, medial amygdalar nucleus, posteroventral part; PAG, periaqueductal gray; PERI, perirhinal area; PL, prelimbic area; PMd, dorsal premammillary nucleus; RSP, retrosplenial area; VMHdm, ventromedial hypothalamic nucleus, dorsomedial part. See text for discussion.

The PMd influences the memory processes that link predatory threats to the associated context. Injection of an NMDA receptor antagonist (2-amino-5-phosphonopentanoic acid) into the PMd during cat odor exposure has been shown to impair conditioned defensive responses to the associated environment (Canteras et al., 2008). Moreover, beta adrenergic blockade in the PMd also reduces contextual fear responses to cat odor (Do Monte et al., 2008). As we shall discuss below, the projection from the PMd to the ventral anteromedial thalamic nucleus (AMv; Canteras and Swanson, 1992; Figure 2A) is a likely pathway to influence mnemonic mechanisms linking predatory threats to their associated context. Recent findings from our group showed that AMv pharmacological inactivation prior to cat exposure did not interfere with innate fear responses but drastically reduced contextual conditioning to the predator-associated environment (De Lima et al., 2013). Thus, the PMd–AMv pathway may be involved in the acquisition of predator-related contextual fear memories. As shown in Figure 2A, the AMv is in a strategic position to convey predator cues to cortico-hippocampal-amygdalar sites involved in the acquisition of contextual fear memories. Previous studies have revealed that the AMv projects to the prelimbic, anterior cingulate, retrosplenial, ectorhinal, and perirhinal cortices as well as to the ventral subiculum and presubiculum (Shibata, 1993; Van Groen et al., 1999). Based on the projection pattern of the AMv, it is clear that this nucleus should exert an important influence on hippocampal processing, either through direct projections to the ventral subiculum and presubiculum or through indirect pathways mediated by the anterior cingulate and retrosplenial areas (Wyss and Van Groen, 1992; Jones and Witter, 2007). The hippocampus is known to be involved in mediating contextual fear memory to predatory threats, and findings from the Blanchard laboratory have shown that ventral hippocampal lesions impair conditioned defensive behaviors associated with either direct exposure to a cat or to its odor (Pentkowski et al., 2006). Moreover, the AMv is also in a position to influence amygdalar sites involved in fear conditioning. As previously noted, the AMv provides moderate input to perirhinal and ectorhinal areas, which are known to provide massive projections to the lateral amygdalar nucleus (Shi and Cassell, 1999). Synapses in the lateral amygdalar nucleus exhibit plasticity that is crucial for fear conditioning (Johansen et al., 2011), and cytotoxic lesions to this region have been shown to block contextual conditioning to predatory threats (Martinez et al., 2011). Taken together, these anatomical findings indicate that the AMv occupies a strategic position to influence both the hippocampus and the lateral amygdalar nucleus, supporting its role in the acquisition of contextual fear memory.

The PMd is also mobilized in response to cat-odor-associated contexts (Staples et al., 2005). Interestingly, there is overlap between the neural systems that integrate unconditioned and contextually conditioned anti-predator defensive responses. Rats exposed to a predator-associated environment exhibit increased Fos expression in elements of the predator-responsive hypothalamic circuit, including the AHN, PMd and, to a lesser extent, the VMHdm (Cezario et al., 2008). As shown in Figure 2B, during exposure to a predator-related context, the AHN is in a position to receive contextual information from the septo-hippocampal system, while the VMHdm is targeted by amygdalar sites involved in predator-related contextual memory. On the efferent side, pharmacological PMd inactivation with the GABA_A_ receptor agonist muscimol blocks conditioned defensive responses during exposure to a predator-associated environment (Cezario et al., 2008).

As for unconditioned anti-predatory defensive responses, the PAG plays a critical role in the expression of contextually conditioned anti-predator responses (Figure 2B). The same pattern of PAG activation observed in response to a live predator, but considerably less intense, was found in animals exposed to an environment previously associated with a predator (Cezario et al., 2008).

## Stimulation of the PMd and dorsal PAG supports fear learning

As previously discussed, the PMd and PAGdl are particularly responsive to predator threats, and we have considered whether stimulation of these sites would be able to mimic internal state changes related to predator threats and therefore serve as a reliable unconditioned stimulus (US) in an olfactory fear conditioning (OFC) paradigm.

The PMd is known to present a substantial plexus of noradrenergic fibers, and beta-adrenoceptor blockade therein results in a clear reduction of innate defensive responses to cat odor. Therefore, we have explored whether beta-adrenergic stimulation of the PMd could serve as a reliable US in an OFC paradigm (Pavesi et al., 2011). Rats were conditioned by pairing the US—an intra-PMd microinjection of isoproterenol (a beta-adrenoceptor agonist)—with the conditioned stimulus (CS)—amyl acetate odor. Beta-adrenoceptor stimulation in the PMd did not produce overt defensive responses but was able to support olfactory conditioning (Pavesi et al., 2011). Accordingly, the subjects that received isoproterenol in the PMd paired with amyl acetate odor as the CS showed marked defensive behavior toward the amyl acetate odor alone 48 h after the conditioning. As described above, the PMd is likely to influence fear learning through a thalamic pathway involving the AMv and its associated cortico-hippocampal-amygdalar circuits. Interestingly, infusion of an NMDA receptor antagonist into another important target of the PMd—the dorsal PAG—was able to block the acquisition of OFC elicited by isoproterenol injection into the PMd (Pavesi et al., 2011), suggesting that the dorsal PAG would also serve for the PMd to provide instructive signals to prosencephalic circuits related to fear learning.

To address the putative role of the dorsal PAG in fear learning, we tested whether glutamatergic activation of the dorsal PAG could serve as a reliable US in an OFC paradigm (Kincheski et al., 2012). Using the same olfactory fear conditioning test described above, rats were subjected to CS/US pairings, with NMDA infusion into the dorsal PAG serving as the US. In contrast to what was observed for PMd beta-adrenergic activation, immediately after the NMDA injection into the dorsal PAG, the rats exhibited flight and jumping behaviors during the first minute of observation. This behavior was followed by an increased amount of freezing, which was limited to the first 5 min (Kincheski et al., 2012). Notably, when the subjects were removed from the conditioning box during the first 5 min of odor exposure, which is when the flight, jumping and freezing responses were predominant, no learning occurred. Conversely, when the rats were removed 5 min after the initial period of vigorous defensive responses, when most of the overt defensive behavior had waned, fear learning was more effective; these results support the idea that NMDA applied directly to the dorsal PAG can serve as a reliable US and is capable of supporting olfactory fear conditioning (Kincheski et al., 2012).

**Figure 3:**
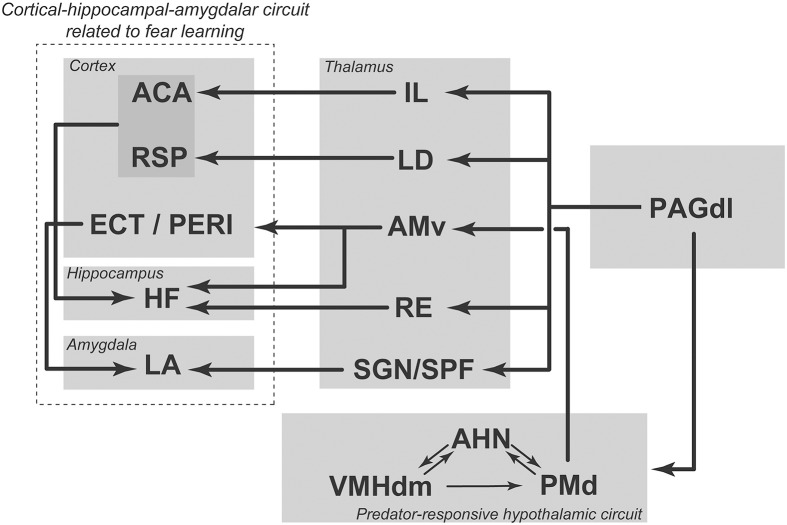
**Summary diagram illustrating ascending projections from the dorsolateral PAG to hypothalamic and thalamic targets; these projections influence cortical-hippocampal-amygdalar circuits related to fear learning**. ACA, anterior cingulate area; AHN, anterior hypothalamic nucleus; AMv, anteromedial thalamic nucleus, ventral part; ECT, ectorhinal area; HF, hippocampal formation; IL, intralaminar nuclei; LA, lateral amygdalar nucleus; LD, laterodorsal thalamic nucleus; PAGdl, periaqueductal gray, dorsolateral part; PERI, perirhinal area; PMd, dorsal premammillary nucleus; RE, nucleus reuniens; RSP, retrosplenial area; SGN, suprageniculate nucleus; SPF, subparafascicular thalamic nucleus; VMHdm, ventromedial hypothalamic nucleus, dorsomedial part. See text for discussion. Modified from Kincheski et al. (2012).

To understand how the dorsal PAG is able to influence prosencephalic circuits related to fear learning, we revisited the ascending connections of the dorsolateral PAG (Kincheski et al., 2012; Figure 3). The main ascending target of the dorsolateral PAG is the anterior hypothalamic nucleus, which, as described above, is part of the medial hypothalamic predator-responsive circuit. In line with this view, electrical stimulation of the dorsolateral PAG up-regulates Fos expression in the PMd (Vianna et al., 2003), supporting the idea that the PAGdl and the predator-responsive circuit operate in concert. Notably, blockade of PMd beta-adrenergic receptors with atenolol has been shown to impair the acquisition of the olfactory fear learning that is promoted by NMDA stimulation of the dorsal PAG (Kincheski et al., 2012). Thus, the projection from the PAGdl to the medial hypothalamic predator-responsive circuit may serve as an important link by which the prosencephalic sites involved in fear learning can be influenced. In addition, as shown in Figure 3, the PAGdl may potentially influence fear learning through a number of parallel thalamic pathways. The PAGdl provides a substantial projection to the intralaminar nuclei. The intralaminar nuclei, in turn, project to the anterior cingulate area, forming a pathway that is involved in fear learning (Furlong et al., 2010). In addition, the PAGdl also projects to a number of other thalamic sites, including the nucleus reuniens, the lateral dorsal nucleus, the suprageniculate nucleus and the parvicellular subparafascicular nucleus, all of which are known to project to elements of the cortical-hippocampal-amygdalar circuit that is involved in fear conditioning, as described above (Van Groen and Wyss, 1992; Linke et al., 2000; Vertes et al., 2006). An important concept emerges from the present analysis: subcortical regions that process elemental fear to predator threats, such as the PMd and the dorsal PAG, may work as critical nodes that serve to instruct prosencephalic sites to promote fear learning. Accordingly, the processing of primal emotional states related to predatory threats should take place in these subcortical nodes and exert a critical influence on fear learning.

## Shock-based fear conditioning using olfactory cues engages elements of the hypothalamic circuit responsive to predator odor

The most common experimental approach to investigate fear processing is shock-based Pavlovian conditioning. However, shock-based fear conditioning to non-olfactory cues does not appear to engage elements of the hypothalamic predator-responsive system and therefore does not seem to be suitable for investigations of the neural basis of fear responses to predator odor (Gross and Canteras, 2012). Accordingly, we have tested whether shock-based fear conditioning to olfactory cues would be a useful system to model predator odor and engage elements of the hypothalamic circuit responsive to cat odor.

**Figure 4:**
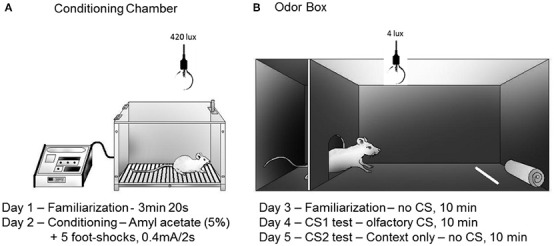
**Schematic drawings representing the conditioning chamber (A) and the odor box (B) used in the olfactory fear conditioning protocol**. Rats were placed inside a stainless steel box located under a fume hood with lighting conditions of 420 lux **(A)** on day 1 (5 min) and day 2 (conditioning). A filter paper saturated with amyl acetate (5%, 250 μl) was used as the olfactory CS. Five electrical foot shocks (0.5 mA, 2 s, 40 s inter-trial interval) were used as the US. The retention of the CS-US association was tested in a Plexiglass box **(B)** that was also located under a fume hood with lighting conditions of 4 lux. The Plexiglass box consisted of a roof-enclosed compartment (left side) and an open (unroofed) compartment (right side). Retention was tested in 10 min sessions on the following three consecutive days (day 3, no-odor, habituation; day 4, olfactory CS exposure test; day 5, associated context, no-odor). The odor source was positioned in the opposite side of the enclosed compartment. The parameters analyzed included the percentage of time spent in the following behaviors: approaching the CS, hiding in the enclosed compartment and stretching out from the enclosed compartment toward the open compartment (head-out). The protocol was based on Dielenberg and McGregor (1999) and Kroon and Carobrez (2009).

Data from our laboratory have confirmed that a neutral olfactory stimulus, such as coffee odor or amyl acetate, can serve as a reliable CS in a fear conditioning paradigm (Canteras et al., 2008). As shown in Figure 4, the experimental paradigm consisted of two consecutive phases: the acquisition of olfactory fear conditioning (days 1 and 2) and the expression of olfactory fear conditioning (days 3–5). The expression of olfactory fear conditioning (second phase) was performed in an odor box (Figure 4) and consisted of three sessions: familiarization (day 3), CS-neutral odor exposure (day 4; test session), and context (day 5). During the familiarization session, the animals did not exhibit fear responses to the odor box, indicating that they did not generalize the fear response to a different context. As the animals were re-exposed to the CS-neutral odor in the odor box, they displayed clear defensive responses and spent most of the time either hiding or engaged in “head-out” behavior. They also avoided approaching the odor source. When the animals were placed in the same context without the CS-neutral odor, the animals exhibited the same sort of defensive behaviors displayed on the previous day during exposure to the CS-neutral odor. These results are important because they demonstrate that the CS-neutral odor was able to mimic a predator odor and produced clear contextually conditioned defensive behavior (Canteras et al., 2008).

Interestingly, the shock-paired neutral odor also up-regulated Fos expression in the PMd. Therefore, similar to cat odor, the shock-paired neutral odor also mobilized the PMd (Canteras et al., 2008). Moreover, the PMd has been shown to be involved in the expression of conditioned responses to a shock-paired neutral odor and to influence the acquisition of CS1-CS2 second-order contextual conditioning. Accordingly, NMDA receptor blockade in the PMd during re-exposure to the footshock-paired neutral odor significantly reduced conditioned fear responses to the CS-neutral odor and resulted in clear impairment of contextual defensive responses on the following day (Canteras et al., 2008).

**Figure 5:**
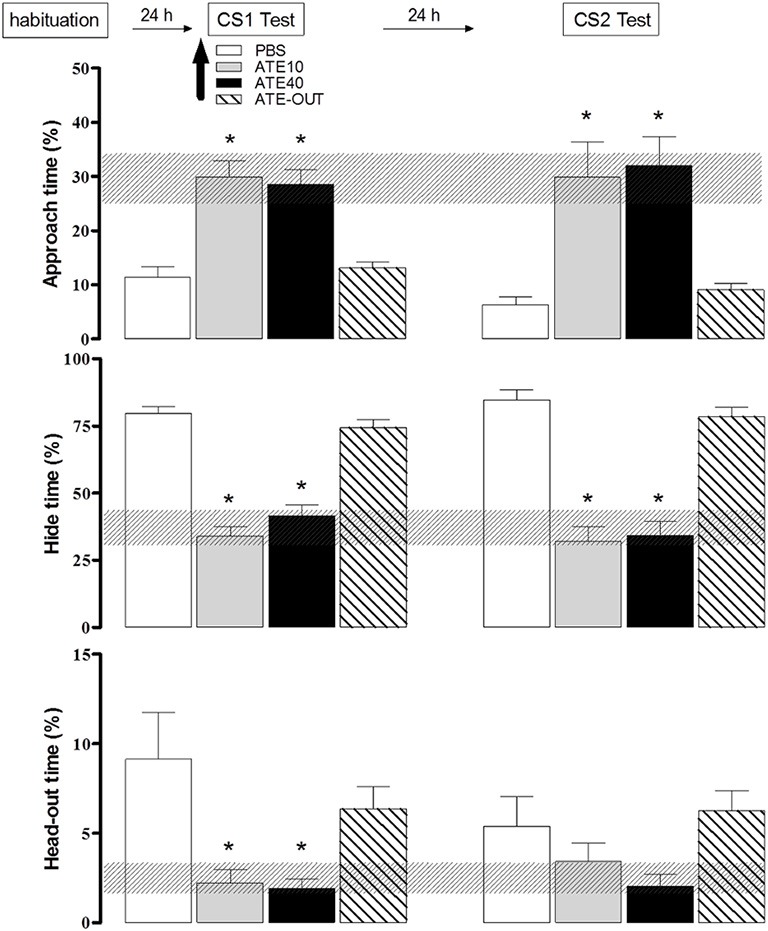
**Effects of dorsal premammillary nucleus (PMd) application of the beta-adrenoceptor antagonist atenolol (ATE; 10–40 nmol; 0.3 μl on the defensive behavior of rats exposed to an olfactory conditioned stimulus (CS)**. The familiarization session and the olfactory (CS1) and context (CS2) sessions (10 min in duration) were conducted over three consecutive days. The parameters analyzed were plotted as the mean (+SEM) and were represented in histograms as the percentage of time spent approaching the odor source (top panel), hiding in the enclosed compartment (middle) and stretching out from the enclosed compartment toward the open compartment (head-out; bottom) during the CS1 or CS2 test session. The hatched horizontal bars represent the confidence limit intervals (within 95%) obtained during the familiarization session. The subjects (*n* = 8–12) received PBS (*n* = 9) or 10 (*n* = 8) or 40 (*n* = 8) nmol ATE, which was administered into the PMd 10 min before the rats were exposed to the CS1 (amyl acetate 5%). ATE OUT (*n* = 12) represents rats in which the cannula was placed outside the PMd. ^*^p < 0.05, compared with the PBS control group (repeated measures ANOVA followed by Newman–Keuls' *post-hoc* test).

We have further tested the role of beta-adrenergic transmission in the PMd to investigate its participation in the expression of shock-based olfactory fear conditioning. Animals were subjected to the shock-based olfactory fear conditioning protocol as previously described. On day 4, prior to the expression of olfactory fear conditioning, the rats were divided into four groups: the control group (PBS), atenolol outside the PMd (ATE-out), and atenolol in the PMD (10 nmol of ATE-10 or 40 nmol of ATE-40). The rats were microinjected into the PMd, and 10 min later, were placed in the apparatus in the presence of the CS-neutral odor. As shown in Figure 5, in contrast to the animals of the other experimental groups (the PBS and the ATE-out groups), the animals in which atenolol was injected into the PMd (the ATE-10 and ATE-40 groups) showed a significant increase in approach time and a significant decrease in hiding and head-out times during exposure to the footshock-paired odor (CS-neutral odor) as well as decreased contextual defensive responses on the following day, as demonstrated by a significant increase in the time the animals spent approaching a neutral cloth and a decreased hide time. Taken together, the experimental data suggest that the PMd may influence both the expression of olfactory fear conditioning and CS1-CS2 second-order contextual conditioning.

**Figure 6:**
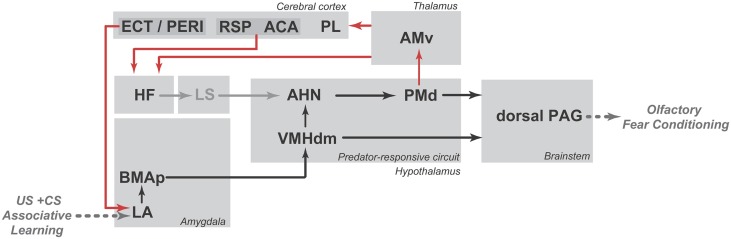
**Schematic diagram showing the putative circuit involved in the expression of olfactory fear conditioning to a neutral odor that was previously paired with footshock (shown in black lines) as well as the PMd–AMv path and the cortical-hippocampal-amygdalar circuit that are putatively involved in second-order contextual conditioning (shown in red lines)**. ACA, anterior cingulate area; AHN, anterior hypothalamic nucleus; AMv, anteromedial thalamic nucleus, ventral part; BMAp, basomedial amygdalar nucleus, posterior part; ECT, ectorhinal area; HF, hippocampal formation; LA, lateral amygdalar nucleus; LS, lateral septum; PAG, periaqueductal gray; PERI, perirhinal area; PL, prelimbic area; PMd, dorsal premammillary nucleus; RSP, retrosplenial area; VMHdm, ventromedial hypothalamic nucleus, dorsomedial part. See text for discussion.

As shown in Figure 6, associative learning between conditioned and unconditioned stimuli is likely to occur in the lateral amygdalar nucleus. In support of this view, tetrodotoxin infusion in the region of the lateral amygdalar nucleus has been shown to impair the acquisition of olfactory fear conditioning (Kilpatrick and Cahill, 2003). Notably, the lateral amygdalar nucleus may influence the medial hypothalamic predator-responsive circuit through its dense projections to the posterior part of the basomedial amygdalar nucleus, which provides substantial input to the ventromedial hypothalamic nucleus (Petrovich et al., 1996). Alternatively, as pointed by Cádiz-Moretti et al. (2014), information about pain stimuli (relayed through the posterior intralaminar thalamic complex and the parabrachial area) and neutral odors (relayed through indirect projections from the piriform cortex and cortical amygdala) may converge in the MEApv, a critical site for responses to predator odors, and this association at the MEApv could easily explain how a neutral odor that was previously paired with foot shock would engage elements of the predator-responsive hypothalamic circuit. However, further studies are needed to provide support for this hypothesis.

As discussed here, the PMd seems to be critical for the expression of olfactory fear conditioning as well as for second-order contextual conditioning. As summarized in Figure 6, the expression of olfactory fear conditioning may depend on PMd projections to the PAG (shown in black lines), whereas second-order contextual conditioning may involve the projection to the AMv and the associated cortico-hippocampal-amygdalar circuits that are related to fear learning (shown in red lines). However, further studies are needed to increase understanding of these pathways and their relationships with olfactory fear conditioning.

## Concluding remarks

Different types of predator odors engage elements of the hypothalamic predator-responsive circuit, which has been primarily investigated in studies using exposure to a live cat or its odor. These studies have provided the general basis of our understanding of how innate and learned fear responses to predator threats are organized. Of particular relevance, primal responses to predator odors involve a neural system formed by the medial amygdalar nucleus, the medial hypothalamic predator-responsive circuit and the dorsal PAG, and elements of this neural system (i.e., the PMd and dorsal PAG) seem to be critical in supplying instructive signals to cortico-hippocampal-amygdalar circuits related to fear conditioning to promote fear learning. According to the present view, the medial amygdalar nucleus processes predator olfactory cues and transmits this information to the medial hypothalamic predator-responsive circuit and the dorsal PAG, where primal emotional states related to predatory threats are processed. This idea provides an interesting perspective of the role of the hypothalamus and dorsal PAG, as opposed to telencephalic sites, in the processing of emotional states in response to predatory threats. Conversely, fear learning depends on instructive signals from these subcortical nodes to cortico-hippocampal-amygdalar circuits for the association of a single cue or contextual information with the emotional state related to the predatory threat.

Interestingly, neutral olfactory stimuli that acquire an aversive valence in a shock-based fear conditioning paradigm may mobilize elements of the hypothalamic-predator responsive circuit, raising interesting possibilities related to how aversive learned olfactory stimuli can mimic predator odor. At this point, future studies are needed to explore how and where associative learning occurs and transforms neutral olfactory stimuli that have been paired with foot shocks into predator-like odors that have the same properties of natural predator odors in inducing innate and learned defensive responses.

### Conflict of interest statement

The authors declare that the research was conducted in the absence of any commercial or financial relationships that could be construed as a potential conflict of interest.
